# Associations between Upper Extremity Motor Function and Aphasia after Stroke: A Multicenter Cross-Sectional Study

**DOI:** 10.1155/2021/9417173

**Published:** 2021-11-09

**Authors:** Shuo Xu, Zhijie Yan, Yongquan Pan, Qing Yang, Zhilan Liu, Jiajia Gao, Yanhui Yang, Yufen Wu, Yanan Zhang, Jianhui Wang, Ren Zhuang, Chong Li, Yongli Zhang, Jie Jia

**Affiliations:** ^1^Department of Rehabilitation Medicine, Huashan Hospital, Fudan University, Shanghai, China; ^2^Xinxiang Medical University, Xinxiang, China; ^3^Department of Rehabilitation Medicine, Shanghai Fourth Rehabilitation Hospital, Shanghai, China; ^4^Department of Neurorehabilitation, The Shanghai Third Rehabilitation Hospital, Shanghai, China; ^5^Department of Rehabilitation Medicine, Shaanxi Provincial Rehabilitation Hospital, Shaanxi, China; ^6^Department of Rehabilitation Medicine, Liuzhou Traditional Chinese Medicine Hospital, Guangxi, China; ^7^Department of Rehabilitation Medicine, The Third Affiliated Clinical Hospital of Changchun University of Chinese Medicine, Jilin, China; ^8^Department of Rehabilitation Medicine, Nanshi Hospital Affiliated to Henan University, Henan, China; ^9^Department of Rehabilitation Medicine, Changzhou Dean Hospital, Jiangsu, China; ^10^Shanghai University of Sport, Shanghai, China; ^11^Fujian University of Traditional Chinese Medicine, Fujian, China; ^12^National Clinical Research Center for Aging and Medicine, Huashan Hospital, Fudan University, China; ^13^National Center for Neurological Disorders, Shanghai, China

## Abstract

**Methods:**

Patients with stroke were compared and correlated from overall and three periods (1-3 months, 4-6 months, and >6 months). Fugl-Meyer assessment for the upper extremity (FMA-UE) and action research and arm test (ARAT) were used to compare the UE motor status between patients with PSA and without PSA through a cross-sectional study among 435 patients. Then, the correlations between the evaluation scale scores of UE motor status and language function of patients with PSA were analyzed in various dimensions, and the language subfunction most closely related to UE motor function was analyzed by multiple linear regression analysis.

**Results:**

We found that the scores of FMA-UE and ARAT in patients with PSA were 14 points ((CI) 10 to 18, *p* < 0.001) and 11 points lower ((CI) 8 to 13, *p* < 0.001), respectively, than those without PSA. Their FMA-UE (*r* = 0.70, *p* < 0.001) and ARAT (*r* = 0.62, *p* < 0.001) scores were positively correlated with language function. Regression analysis demonstrated that spontaneous speech ability may account for UE motor function (*R*^2^ = 0.51, *p* < 0.001; *R*^2^ = 0.42, *p* < 0.001). Consistent results were also obtained from the analyses within the three time subgroups.

**Conclusion:**

Stroke patients with PSA have worse UE motor performance. UE motor status and language function showed positive correlations, in which spontaneous speech ability significantly accounts for the associations.

## 1. Introduction

Patients after stroke who have both upper extremity (UE) motor impairment and/or language dysfunction are common [1]. These two types of poststroke dysfunction are the most apparent neuropsychological deficits occurring after stroke: UE motor deficit occurs in about 80% of stroke survivors, aphasia in 21%-38%, and cooccurrence in about 24% [2–4]. PSA with UE motor dysfunction impacts social participation and quality of life, and it can also be associated with multiple comorbidities and lead to worse prognosis [5, 6]. Due to the adjacent anatomical location, ischemia or hemorrhage in the middle cerebral artery (MCA) often leads to UE motor dysfunction and nonfluent aphasia. Nevertheless, there are small samples of study that have analyzed the relationship between hand-arm motor dysfunction and aphasia using lesion volume and location as control variables, showing that the association is not determined by anatomical relationships alone. The extents and limitations of UE and language cortical reciprocity remain under debate; it is likely that UE movement and language have shared neural correlates not merely depending on anatomical proximity and vascular factors.

In Huashan Hospital, there is an original operation, a contralateral seventh cervical nerve transfer to improve UE motor function in patients with chronic central injury [7]. After the surgery, we found that patients with PSA not only improved their UE motor status but also their language function. These phenomena suggest a deep neural mechanism relationship between language function and UE motor status after stroke. Several studies [8–11] have focused on the potential relationship between UE motor status and language function. From a human evolution perspective, language was spurred by freedom of hand movement as an additional consequence of this upright posture. Gestures are a combination of UE movements and language [12]. A retrospective cohort study addressed the possible interaction between motor impairment and aphasia recovery after stroke. Motor responders showed better linguistic performances at the final aphasia assessment than motor nonresponders, while language responders reached a higher level of motor functioning than language nonresponders [8]. Meanwhile, a significant response in one domain was not associated with any deterioration in the other. Furthermore, Harnish et al. examined five patients with aphasia and hemiparesis poststroke during six weeks of UE therapy but not receiving speech therapy. Patients were assessed not only for the UE motor recovery but also for changes in their language abilities. fMRI data demonstrated shifts in increased blood oxygen improvements in both UE motor status and language function scores [13]. However, current studies rarely focus on simultaneous UE motor dysfunction with language deficits and even less on both functions' concurrent recovery during stroke recovery. Most studies only unexpectedly found this phenomenon or were mostly exploratory paradigm intervention studies [1, 14–16]. Few studies have focused on the difference in UE motor function status between patients with PSA and nonphasic poststroke patients. Moreover, no study provides evidence on the correlation between UE motor status and language function after stroke [8, 9], which leads to low attention to UE-language correlation so that UE and speech-language therapies are completely separated during UE motor and (or) speech rehabilitation.

To cover this gap, the present study investigated the UE motor status and language function of stroke patients by a cross-sectional investigation. We hypothesized that there were differences in motor status between stroke patients with PSA and without PSA and that there were some relationships between the speech-language function and UE motor status in patients with PSA.

Therefore, the objectives of this study were (1) to compare the UE motor status between patients with PSA and without PSA, (2) to investigate the association between language function and UE motor status in patients with PSA, and (3) based on (2), to determine which dimension of PSA evaluation is most closely related to the UE motor status.

## 2. Methods

### 2.1. Study Population and Design

This study was conducted between May 2020 and June 2021 in the departments of rehabilitation medicine of six hospitals from different regions in China. Patients were consecutively screened for the following criteria: (1) aged 18 years or older; (2) native Chinese speaker, (3) stroke onset > 1 week, (4) with a primary diagnosis of acute cerebrovascular accident according to the WHO diagnostic criteria confirmed by computed tomography (CT) or magnetic resonance imaging (MRI), (5) underwent rehabilitation assessed by a team of specialists (physicians, speech therapists, and occupational therapists), and (6) had ability to complete all the assessment. However, individuals were excluded if the consent of the patient's family could not be obtained; if there was no imaging available; if they had a previous history of stroke; if they had a severe hearing impairment or visual impairment; if they had other primary medical conditions that could influence language and motor function; such as a brain tumor, Parkinson's disease, severe poststroke depression, and Alzheimer's disease; or if they had undergone surgical evacuation.

Patients were evaluated in a single test session performed by speech therapists and occupational therapists who had received consistency training. One trained researcher performed the data collection. Patients' baseline characteristics were evaluated, including age at stroke onset, gender, comorbidities, hand dominance, time poststroke, lateralization, and stroke type. After 2326 patients were screened, those who met the above conditions participated in this study, 214 among whom with PSA were in the observational PSA group. A group of 221 patients without PSA after stroke matched for age and sex participated and were distributed into the non-PSA group as controls. The sample sizes were estimated referring to other similar studies [8, 17, 18]. As an important outcome, the UE motor impairment and function between the two groups were compared. Further evaluation was done in the observational PSA group to see the association between UE motor status and language function evaluation scores. For further validation purposes, the relationships between them were analyzed by multiple linear regression. Then, to observe the difference between different time periods from stroke onset, subsequent stratification analyses by time (1-3 months, 4-6 months, and >6 months) were performed. Our study used a cross-sectional observational design. The ethics committee approved the study protocol of Huashan Hospital of Fudan University and all participating centers according to the 1964 Declaration of Helsinki's ethical standards and its later amendments. This trial is registered with ChiCTR2000033792. All patients or their families provided written informed consent before study enrollment.

### 2.2. Measurement Instruments and Evaluation

#### 2.2.1. Evaluation of PSA: Aphasia Quotient of Western Aphasia Battery-Revised (WAB-AQ) and Boston Diagnostic Aphasia Examination (BDAE)

PSA was evaluated using the Chinese version of WAB-AQ, a commonly used clinical evaluation of PSA that assesses the presence, type, and severity of aphasia with a 0-100 scale (score < 93.8 are indicative of aphasia). The WAB-AQ elaborately evaluates the domains of expression and comprehension, yielding summary scores for the following four domains: spontaneous speech, auditory verbal comprehension, repetition, and naming. The four dimensions of scores were recorded and counted. AQ, the weighted composite of these four scores, was used as the independent variable of interest in this study and is indicative of the overall severity of the patients' PSA. On the other hand, for easy screening and observation, the BDAE severity grading standard was chosen to classify the severity of patient language dysfunction with grade criteria of 0, 1, 2, 3, 4, and 5 [19]. Grade 0 is meaningless language or auditory comprehension, while grade 5 is a barely recognizable language disorder, and the patient may have some subjective difficulties, but it is not easy for the listener to detect. All patients have to be assessed by BDAE, and only if the grade < 5 will WAB-AQ be evaluated.

#### 2.2.2. Evaluation of UE Motor Impairment: Fugl-Meyer Assessment for the Upper Extremity (FMA-UE)

The FMA was used to assess extremity motricity, balance, some sensory details, and joint dysfunction in hemiplegic patients. We evaluated the only motor function of the UE, including measurement of voluntary movement, velocity, coordination, and reflex activity. A total of 33 items are included. A 3-step (0-1-2) ordinal scale is applied to each item (0 = details cannot be performed; 1 = details are performed only partly; 2 = details are performed throughout the full range of motion of the joint). This gives a total maximum score of 66, which defines a normal motor function (42 and 14 for the arm and hand, respectively). FMA-UE mainly aims at evaluating UE motor impairment and dysfunction after stroke.

#### 2.2.3. Evaluation of UE Motor Function: Action Research Arm Test (ARAT)

Instruments needed to perform the test are as follows: woodblocks, a ball, a washer and bolt, a stone, two different sizes of alloy tubes, two glasses, a marble, and a 6 mm ball bearing (instrument model: OT-KL-40400). The test is a 4-grade scale ranging from 0 to 3 with a maximum score = 57 (0 = can perform no part of the test; 1 = can perform the test partially; 2 = can complete the test but takes an abnormally long time or has great difficulty; and 3 = can perform test normally). ARAT is a quantitative test for the UE function and includes four subsets: grasp, grip, pinch, and gross movement. Both ARAT and FMA-UE are widely used and are the most recognized methods to evaluate the motor status of UE in patients with stroke. The difference is that ARAT is mainly aimed at motor function assessment and activity measurement, while FMA-UE pays more attention to dysfunction and impairment.

### 2.3. Statistical Analysis

Data were analyzed with IBM SPSS Statistics version 26.0. Demographics and clinical variables, presented as mean ± standard deviation for continuous variables and proportions for categorical variables, were compared between observation and control groups using the independent sample Student *t*-test, the Chi test, and the Mann–Whitney *U*-test, as appropriate. The Spearman correlation analysis between WAB-AQ and FMA-UE scores was made to address the association question. Then, to eliminate the influence of some factors on the correlation analysis, the correlation coefficients between WAB-AQ and FMA-UE are corrected for age, education, and duration poststroke. Similarly, this method is also used between WAB-AQ and ARAT scores and between the four parts of WAB-AQ (spontaneous speech, understanding, repetition, and naming) and FMA-UE as well as ARAT scores. In addition, all patients were stratified according to 3 time periods (1-3 months, 4-6 months, and >6 months) and compared, and correlated analyses were performed within each of the three periods by the same method as the overall analysis. In the end, we performed two multiple linear regression analyses, using the “enter” method, to determine which dimension of WAB-AQ was the most informative in accounting for the UE motor function with WAB-AQ including spontaneous speech, comprehension, repetition, and naming scores as independent variables and ARAT or FMA-UE scores as dependent variables.

## 3. Results

### 3.1. Demographics

**Figure 1:**
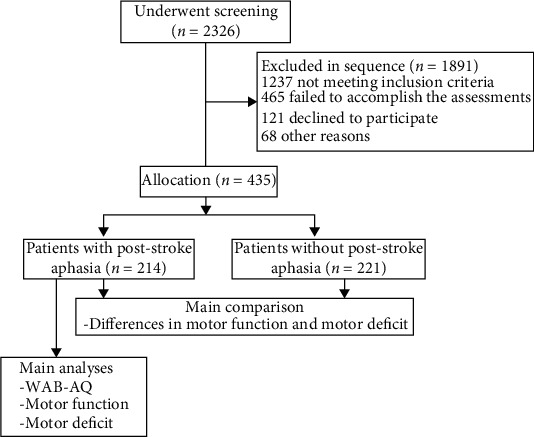
Flow chart of the study sample and procedures of the comparison and analyses. WAB-AQ indicates the Western Aphasia Battery-Aphasia Quotient; FMA-UE indicates the Fugl-Meyer assessment for the upper extremity; ARAT indicates the action research and action test.

From a total of 2326 patients, we excluded 1891, leaving 435 patients for analysis (see Figure 1). 435 patients underwent a complete systematic assessment with a median course of 15 weeks (IQR: 7-32). The median age of the patients was 60.6 years (SD = 11.2). A total of 153 were female, and 282 were male.  Table 1(a) shows the patient characteristics, presented for the total group and for the patients with PSA (*n* = 214, 49.2%) and without PSA (*n* = 221, 50.8%). 370 patients suffered from ischemic stroke and 65 from hemorrhage. A total of 330 patients showed right-sided hemiparesis, while 105 patients showed left-sided hemiparesis. Stratification according to stroke duration showed 69 in the PSA group and 76 in the non-PSA group for patients stratified according to a period of 1-3 months; 69 in the PSA group and 72 in the non-PSA group for patients stratified according to a period of 4-6 months; and 76 in the PSA group and 73 in the non-PSA group for patients stratified according to a period >6 months. No statistically significant difference was found in age, gender, comorbidities, hand dominance, time poststroke, and type of stroke between groups.

### 3.2. Comparison between Groups and Distribution of PSA Group

**Figure 2:**
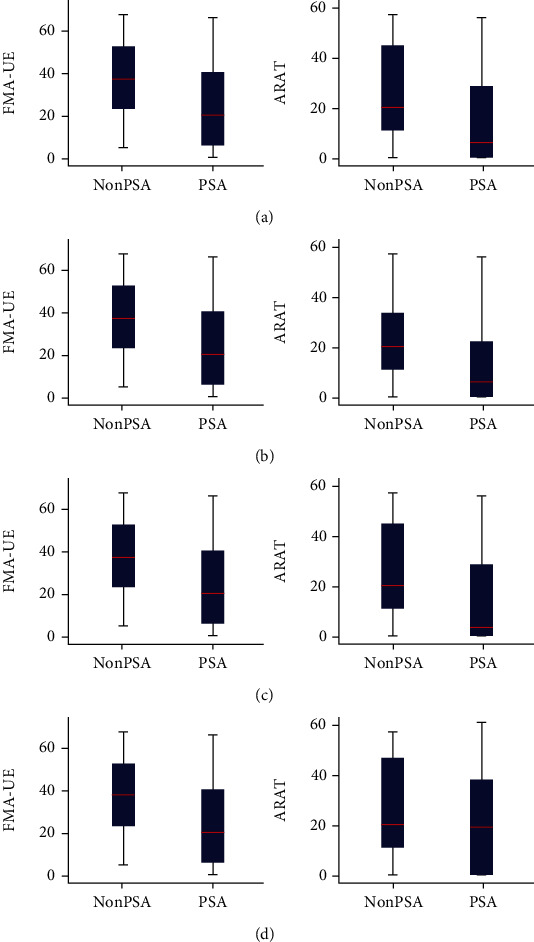
Clinical measurement of the FMA-UE and ARAT scores. (a) Comparison of the FMA-UE and ARAT total scores between the non-PSA and PSA groups. (b–d) Comparisons of the FMA-UE and ARAT total scores between the non-PSA and PSA groups in 1-3 months, 4-6 months, and >6 months. Significant differences were observed in both groups. *p* < 0.001. Abbreviation: non-PSA indicates patients without poststroke aphasia; PSA indicates patients with poststroke aphasia; FMA-UE indicates the Fugl-Meyer assessment for the upper extremity; ARAT indicates the action research and action test.

The FMA-UE and ARAT scores were compared between groups through the Wilcoxon rank sum test. The confidence interval estimation of median difference based on the Wilcoxon rank sum test is obtained by the Hodges-Lehmann method. The contrast revealed a significant difference between groups (*p* < 0.001; Table 1(b)), and it showed that the non-PSA group had significantly higher scores than the PSA group (*p* < 0.001, Figure 2). Detailed scores of the four dimensions in the 214 PSA patients are summarized in Table 1(b). After stratification according to the stroke time, the three comparisons (PSA versus non-PSA) of subgroups (1-3 months, 4-6 months, and >6 months) still obtained consistent results (*p* < 0.001, Figure 2).

### 3.3. Correlations between PSA and Motor Function and Deficit

**Figure 3:**
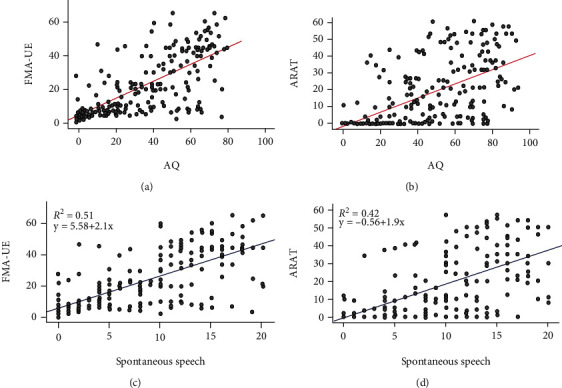
Correlation and regression in independent evaluation scores. (a, b) The association of AQ with FMA-UE and ARAT is shown. (c, d) The correlation of spontaneous speech and FMA-UE and ARAT is shown using linear regression equation. *p* < 0.001. FMA-UE indicates the Fugl-Meyer Assessment of the Upper Extremity; ARAT indicates the action research and action test; AQ indicates the Western Aphasia Battery-Aphasia Quotient.

**Figure 4:**
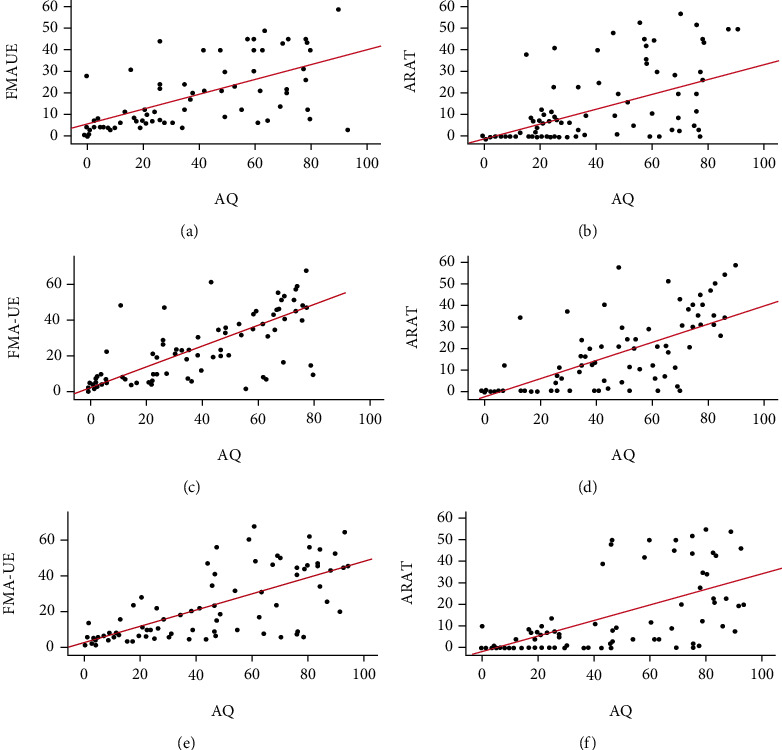
Correlations of independent evaluation scores in different times from stroke onset. (a, b) The association of AQ with FMA-UE and ARAT in 1-3 months is shown. (c, d) The association of AQ with FMA-UE and ARAT in 4-6 months is shown. (e, f) The correlation of AQ with FMA-UE and ARAT in >6 months. *p* < 0.001. FMA-UE indicates the Fugl-Meyer assessment of the upper extremity; ARAT indicates the action research and action test; AQ indicates the Western Aphasia Battery-Aphasia Quotient.

Table 2 illustrates the results of the correlation analyses between language functions (WAB-AQ, spontaneous speech, comprehension, repetition, and naming score) and UE motor status (FMA-UE and ARAT scores) from the overall perspective and from the perspective of the three time periods. We adjusted the correlation coefficients with age, education, and duration poststroke. Overall, moderate to strong positive correlations were found between WAB-AQ and ARAT score (*r* = 0.62, *p* < 0.001, Figure 3(b)). Further, there were stronger correlations between WAB-AQ and FMA-UE score (*r* = 0.70, *p* < 0.001, Figure 3(a)). For all the factors analyzed, their correlation coefficients varied from 0.45 to 0.72, of which the weakest correlation was comprehension, and the strongest was spontaneous speech. All results of partial correlation analysis, taking age, education, and duration poststroke as covariates, are shown in Table 2. Consistent with overall correlation results, the results of the partial correlation analyses according to the time stratification are shown in Table 2. We found that the highest correlation coefficient was WAB-AQ and FMA-UE in 4-6 months (*r* = 0.76, *p* < 0.001). Overall, the time stratification association trends were consistent with the overall analyses (see Figure 4).

### 3.4. Factors Associated with Motor Dysfunction

Two multiple linear regression analyses were performed to identify the most related factors that affect ARAT and FMA-UE scores. In the first regression model between four variables of WAB-AQ (spontaneous speech, comprehension, repetition, and naming score) and ARAT score, the results demonstrated that the four independent variables of WAB-AQ explained 42% of the variance in the ARAT score (*R*^2^ = 0.42, *p* < 0.001). However, only the spontaneous speech score was significant (*R*^2^ = 0.42, *p* < 0.001, Figure 3(d)). The other three variables had no significant difference (*p* > 0.05). The second regression model also examined the four independent variables with the FMA-UE score. The results demonstrated that the four independent variables of WAB-AQ explained 51% of the variance in the FMA-UE score (*R*^2^ = 0.51, *p* < 0.001). Similarly, only the spontaneous speech score was significant (*R*^2^ = 0.51, *p* < 0.001, Figure 3(c)); the other three variables had no significant difference (*p* > 0.05).

## 4. Discussion

Our results demonstrated that the UE motor status of patients without PSA is better than those with PSA, and there are positive relationships between UE motor status and language functions in patients with PSA (see Table 2). Spontaneous speech ability, one of the language functions, is most closely related to UE motor status, which explained 51% of the variance in the motor deficit and 42% in motor function, respectively (see Figures 3(c) and 3(d)). Previous studies have mentioned that the recovery of motor and language function is operated in parallel [20–23]. Due to the lack of data demonstrating UE motor status associated with language function, current stroke rehabilitation evaluations and therapies have treated these two symptoms separately [8, 24]. Patients with PSA receiving speech-language therapy are frequently seated during treatment, with UE impassive and motionless [9]. Our results supported the hypothesis that poststroke patients' UE motor status and language function are highly correlated, and UE motor status assessment and therapy should be integrated into the treatment for patients with PSA [9].

Similar to the findings of previous studies [25, 26], after evaluation of FMA-UE, ARAT, and WAB-AQ, we found that the evaluation scores of patients with PSA were significantly lower than those of nonaphasia patients with no difference in age, educational background, and course of stroke between the two groups not only from an overall perspective but also from three time perspectives (see Figure 4). PSA is independently associated with increased complications and length of stay during the acute stroke admission after controlling for NIHSS score, with an effect comparable to severe hemiparesis, and sometimes greater [26]. Likewise, patients with PSA have lower motor Functional Independence Measures (FIM) and cognitive FIM scores both at admission and at discharge, compared to those without PSA during the subacute and chronic period [25]. Our findings support their findings and provide a supplement and explanation for this phenomenon. FIM is a routine assessment in stroke rehabilitation centers to quantify the ability to perform daily activities after stroke with a 7-point scale for 5 cognitive and 13 motor tasks such as getting dressed, bowel, and grooming control [10]. FMA-UE and ARAT scales are specifically used to evaluate UE motor deficit and motor function for stroke patients [20]. Overall, our results provide preliminary evidence why aphasia patients have worse FIM scores and long hospitalization.

**Figure 5:**
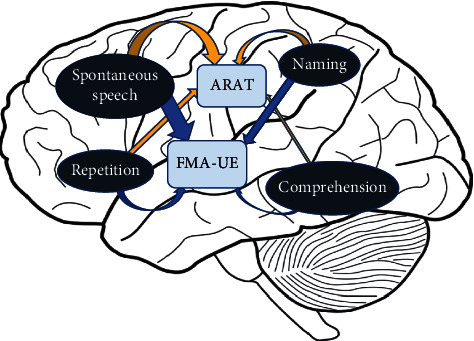
The association between UE motor status and language function after PSA. Schematic diagram shows partial correlations in an eight-way analysis of FMA-UE; ARAT; and spontaneous speech, comprehension, repetition, and naming. The degree of the arrow thickness between two modules is proportional to the correlation coefficient. FMA-UE indicates the Fugl-Meyer assessment for the upper extremity; ARAT indicates the action research and action test.

Hybbinette et al. [27] confirmed the common occurrence of apraxia of speech and aphasia in left hemisphere stroke patients with a hand motor impairment through a small sample study. Our correlation analyses results show that the four dimensions of language function—spontaneous speech, comprehension, repetition, and naming—were all associated with UE motor status (see Figure 5). The correlation between spontaneous speech and UE motor status is the strongest, while the correlation of comprehension is the weakest among the four dimensions. Because some patients have been paralyzed for a long time, the ARAT scale has basic requirements for the UE function. Some of the patients had low or even zero scores of ARAT, which reduce the correlation coefficient to a great extent (seeing Figure 3(b)). Furthermore, regression analyses show that spontaneous speech ability can account for UE motor status to some degree. Consistent with previous studies, our results make their conclusions more convincing that the Aachen aphasia test (AAT) is a predictor of functional outcome in patients with aphasia [26]. Its predictive power is like that of other functional tests commonly recognized to predict outcome strongly. Among the language functions in AAT, comprehension seems to be the most important predictive factor of the total and cognitive FIM, while spontaneous speech ability seems to be a motor-FIM predictor. There were unexpected findings in previous studies that in the treatment of UE motor deficits, the patient's language function was improved, or when the PSA was treated, the UE motor function was improved [15, 28–31]. For example, transcranial direct current stimulation (tDCS) is utilized to stimulate the left primary motor cortex (M1) to study its effect on language function. To explore its clinical effect, some researchers used M1-tDCS to intervene in patients with PSA. The results show that M1-tDCS can improve aphasia patients' motor and communication function in conjunction with enhancing the retrieval ability of action-related words in the long term [16]. Interestingly, studies demonstrated that language function could be improved by asking patients to watch videos of task-oriented movements of the UE with voice guidance [31]. Similarly, compared with the control group, some movements such as grip without phonetic guidance can also enhance patients' language function with PSA. However, the extent and limitations of UE and speech-language cortical reciprocity remain unclear, and whether the affected anterior brain regions of the language-dominant hemisphere are interwoven with proximate cortical areas supporting UE motor status [24].

Our results provide compelling evidence for the relation between UE motor status and language function in terms of behavioral performances and demonstrate that this relationship can be applied to patients' therapy with PSA or UE motor deficit or both after stroke. Patients with PSA have worse hand and UE motor status, which calls for more attention to be given to UE motor rehabilitation in these patients. Interactions between the auditory system and the motor system are related to speech perception. The motor theory of perception has two basic claims: perceiving speech is perceiving gestures and perceiving speech involves the motor system. The mirror neuron system (MNS) is a multimodal system composed of neuronal populations that respond to motor, visual, and auditory stimulation, such as when an action is performed, observed, heard, or read about. In humans, the MNS has been identified using neuroimaging techniques. It reflected the integration of motor-auditory-visual information processing related to aspects of language learning, including action understanding and recognition [32]. Based on MNS, embodied cognition theory believes that various cognitive processes (such as concepts, categories, language, reasoning, and judgment) are closely related to the body's sensorimotor system [33, 34]. Therefore, the realization of language processing should take advantage of the brain motor network, that is, the interweaving and coupling of language processing and motor execution [35]. These theories can demonstrate our findings from the aspect of neural mechanisms.

Our study has some limitations. We did not classify patients according to recovery stage, severity, and the specific brain damage area in patients. Moreover, our study was performed in the cross-section without longitudinal follow-up; thus, whether the recovery stage affects their correlations is unclear. Furthermore, given the proximity of hand-arm and speech-language neural structures, in many patients with poststroke aphasia, the contralesional UE is often simultaneously impaired so that the association between them seems inevitable [9]. However, we know that the Broca area (BA44,45) is adjacent to the UE motor cortex, which is mainly responsible for spontaneous speech ability. Nevertheless, in addition to spontaneous speech, naming, repetition, and comprehension are also positively associated with UE motor conditions, and there should be a deeper neural mechanism worth exploring. Another limitation is that although our study has a large sample size, this also led to a less strict implementation of our inclusion and exclusion criteria, where some of the patients may have been accompanied by other symptoms after stroke. In addition, the fact that the patients were not specifically restricted in terms of damaged brain location and only excluded some patients with large brain lesion only, also diminished the persuasiveness of our findings, and we will go on to restrict these factors in our next study and try to get more rigorous conclusions.

## 5. Conclusion

To our knowledge, this is the first cross-sectional study to explore the relationship between UE motor status and language function after stroke. Our study demonstrated that patients with PSA tend to be with poorer UE motor status compared to those without PSA, and UE motor status is positively correlated with language function, especially for spontaneous speech ability. Future study should focus more on the deeper mechanisms of the link between UE motor status and language function after strictly controlling the location and severity of brain lesion. In addition, this study provides a new perspective and statistical evidence for a “combined assessment and therapy” approach to UE motor and speech-language rehabilitation, which remains to be demonstrated in future studies.

## Figures and Tables

**Table:** (a) Comparisons of demographic data between the PSA group and the non-PSA group

	PSA group (*n* = 214)	Non-PSA group (*n* = 221)	*p* value
Female, *n* (%)	82 (38.3)	71 (32.1)	0.176^b^
Age, mean (SD) (y)	61.1 ± 11.9	60.1 ± 10.4	0.340^a^
Education, mean (SD) (y)	10.32 ± 3.7	10.91 ± 5.4	0.184^a^
Duration poststroke, median (IQR) (week)	16 (6-35)	14 (7-32)	0.316^c^
Type of injury, *n* (%)			0.586^b^
Ischemia	180 (84.1)	190 (86.0)	
Hemorrhage	34 (15.9)	31 (14.0)	
Affected limb, *n* (%)			0.297^b^
Right	167 (78.0)	163 (73.8)	
Left	47 (22.0)	58 (26.2)	

**Table:** (b) Comparisons of clinical variables between the PSA group and the non-PSA group

	PSA group (*n* = 214)	Non-PSA group (*n* = 221)	Mean difference (95% CI)	*p* value
Motor evaluation, median (IQR)				
FMA-UE	20 (7-40)	35 (23-52)	14 (10, 18)	<0.001^c^
ARAT	5.5 (0-30)	21 (11-45)	11 (8, 13)	<0.001^c^
Language evaluation, median (IQR)				
BDAE	1.57 ± 1.18	5	-3.43 (-3.6, -3.3)	<0.001^d^
WAB-AQ	44.6 (18.1-70.6)			
Spontaneous speech	7.0 (2.0-13.0)			
Comprehension	126.0 (60.0-175.0)			
Repetition	50.0 (9.8-80.0)			
Naming	27.0 (0.8-67.3)			

^a^Two independent sample *t*-test. ^b^*χ*^2^ test. ^c^Wilcoxon's rank sum test. ^d^Single sample *t*-test. Abbreviations: SD indicates standard error of the mean; IQR indicates interquartile range; CI indicates confidence interval; FMA-UE indicates the Fugl-Meyer assessment of the upper extremity; ARAT indicates the action research and action test; BDAE indicates the Boston Diagnostic Aphasia Examination; WAB-AQ indicates the Western Aphasia Battery-Aphasia Quotient.

**Table 2:** Pearson's correlation between four parts of WAB-AQ and FMA-UE and ARAT scores.

*r*					
Language motor	WAB-AQ	Spontaneous speech	Comprehension	Repetition	Naming
FMA-UE^†^	0.70	0.72	0.53	0.60	0.64
ARAT^†^	0.62	0.66	0.45	0.52	0.57
FMA-UE^∗^	0.59	0.60	0.46	0.52	0.46
ARAT^∗^	0.54	0.60	0.42	0.42	0.45
FMA-UE^∗∗^	0.76	0.76	0.57	0.65	0.72
ARAT^∗∗^	0.68	0.68	0.48	0.57	0.67
FMA-UE^∗∗∗^	0.71	0.76	0.52	0.62	0.67
ARAT^∗∗∗^	0.65	0.70	0.46	0.58	0.61

^†^Correlation analyses of the overall time period. ^∗^Correlation analyses of 1-3 months. ^∗∗^Correlation analyses of 4-6 months. ^∗∗∗^Correlation analyses of >6 months. FMA-UE indicates the Fugl-Meyer assessment for the upper extremity; ARAT indicates the action research and action test; WAB-AQ indicates the Western Aphasia Battery-Aphasia Quotient. *p* < 0.001. The correlation coefficients are corrected for age, education, and duration poststroke.

## Data Availability

Data are available from the corresponding author on reasonable request.

## Abbreviations

UE: Upper extremity
PSA: Poststroke aphasia
FMA-UE: Fugl-Meyer assessment for the upper extremity
ARAT: Action research and action test
WAB-AQ: Western Aphasia Battery-Aphasia Quotient
BDAE: Boston Diagnostic Aphasia Examination.
