# Supplementary material for: Associations between Upper Extremity Motor Function and Aphasia after Stroke: A Multicenter Cross-Sectional Study
Source: Behav Neurol. 2021 Nov 9;2021:9417173. doi: 10.1155/2021/9417173 (PMC8595012; doi:10.1155/2021/9417173)
Supplement: Supplementary Materials — Details of functional assessment scales can be found in the supplemental file. [file 9417173.f1.docx]

**No.1 The Basic Information**

**Name:** Male/Female Age:

**Date of onset:**

**Occupation: Education** (years of education):

**Hand-handed:** Left / right

**CT** (location, nature, size):

**MRI** (location, nature, size):

**Diagnosis of disease** (cerebral infarction/cerebral hemorrhage):

**Language Diagnosis** (severity):

**Paralyzed side**: Left/right **Brunnstrom grading**: Hand Upper Limb Lower Limb

**ADL:**

**Sensory impairment**: shallow/dark left/right upper/lower limb

**Tendon reflexes**: upper left/right up/down/weak

**Pathological reflexes**: left/right /-

**Dysphagia**: with/without

**Oral surface dysfunction:** extension of the tongue, swelling of the cheek, grinning, vibration of the tongue

**Basic diseases:**

**No.2 Boston Diagnostic Aphasia Examination Severity grading**

| grading | Language expression | Year Month Day | Year Month Day | Year Month Day |
| --- | --- | --- | --- | --- |
| Level 0 | No meaningful speech, no auditory comprehension. |  |  |  |
| Level 1 | There are discontinuous verbal expressions in speech communication, but most of them require the listener to speculate, ask and guess; |  |  |  |
| Level 2 | With the help of the listener, a conversation about familiar topics may take place. But unfamiliar topics often fail to express their thoughts, making it difficult for patients and examiners to communicate verbally. |  |  |  |
| Level 3 | Patients can discuss almost all of their daily problems with little or no help. However, the weakening of speech and/or comprehension makes certain conversations difficult or unlikely. |  |  |  |
| Level 4 | Speech is fluent, but there are observed impairments of understanding, but there are no obvious limitations in thought and speech expression. |  |  |  |
| Level 5 | There are very few identifiable speech disorders that patients feel subjectively difficult, but the listener may not be visibly aware of them. |  |  |  |

**No.3 Western Aphasia Battery**

1. **The amount of information** in spontaneous speech **(out of 10 points),** **fluency, grammatical ability, and missteps** **(out of 10) is checked by a total score of 20 points**

**Amount of spontaneous speech information (scoring criteria see III)**

| II. Issues | | | finish | features | remark |
| --- | --- | --- | --- | --- | --- |
| (1) How are you today? | | |  |  |  |
| 2 Have you been here before? | | |  |  |  |
| What's your name? | | |  |  |  |
| (4) Where do you live? | | |  |  |  |
| (5) What do you do? | | |  |  |  |
| (6) Why are you here? | | |  |  |  |
| (7) Please tell me, what do you see in this picture? Try telling me the sentence | | |  |  |  |
| summary | Information volume score: | Fluency, grammar, and misstation scores: | | | |

**Fluency assessment chart (allowing subjects to describe the image; rating criteria see IV).**


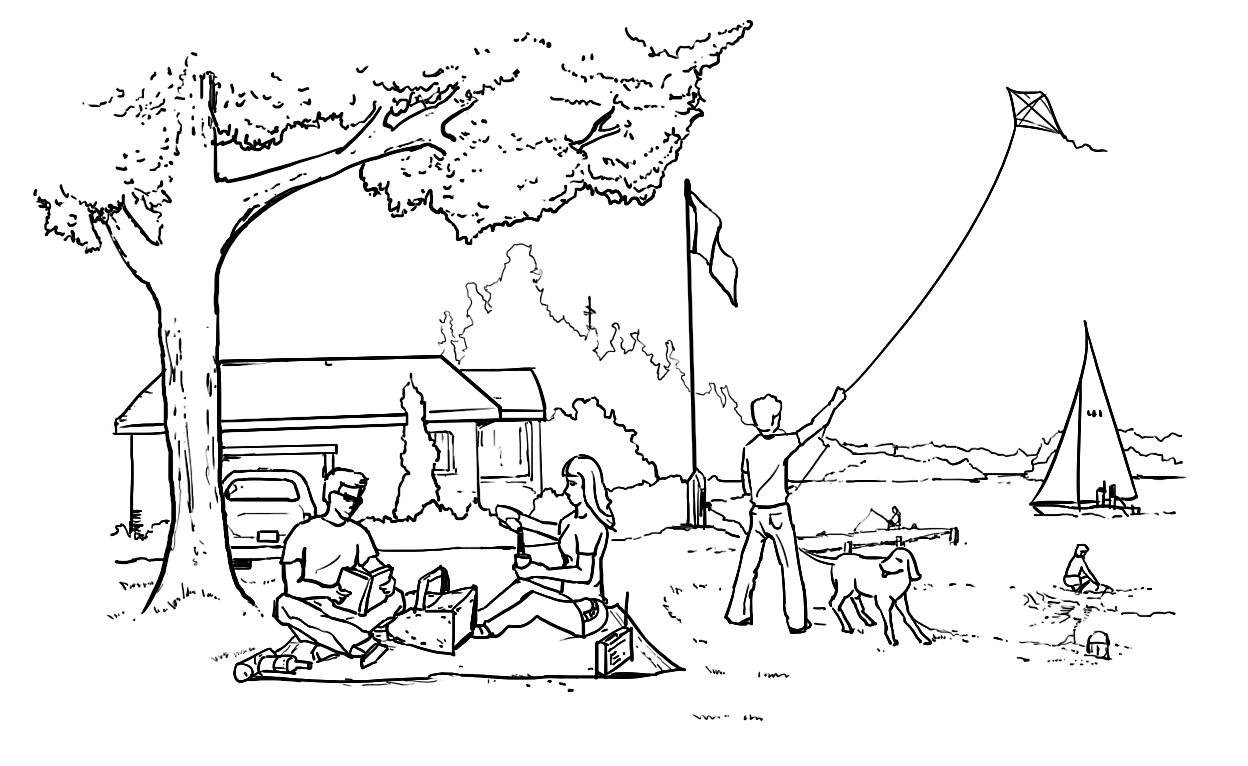


| **III. Scoring criteria** |
| --- |
| 0 points: No information at all |
| 1 point; only incomplete reactions, such as uttering only the last name or first name |
| 2 points: Of the first 6 questions, only 1 answered correctly |
| 3 points: Of the first 6 questions, only 2 were correct |
| 4 points: 3 of the first 6 questions were correct |
| 5 points: 3 of the first 6 questions were correctly answered and there were some reactions to the picture |
| 6 points: 4 of the first 6 questions were correct and there were some reactions to the picture |
| 7 points: Of the first 6 questions, 4 were correctly answered, and there were at least 6 descriptions of the picture |
| 8 points: 5 of the first 6 questions were correct, and the picture was not fully described |
| 9 points: in the first 6 questions, all the answers are correct, the picture can be almost completely described, that is, can name at least 10 characters or actions, there may be a back-and-forth statement |
| 10 points: The first 6 questions are completely correct, with normal length and complex sentences to describe the picture, and a reasonable and complete description of the picture |
|  |
| **IV. Check scoring criteria for fluency, grammar, and missteps** |
| 0 points; completely wordless or only short and meaningless words |
| 1 point: repeated stereotyped words in different tones, have some meaning |
| 2 points: Say a few individual words, often with misstatement, effort and hesitation |
| 3 points: smooth repetitive words or grunts, with very little chic (jargon) |
| 4 points: Sage, telegraphic speech, mostly words that mean a single word, often have the wrong word, but occasionally verbs and prepositional phrases, only spontaneous words such as "Oh, I don't know" |
| 5 points: telegraphic, some grammar structure of the more fluid language, misstatement is still obvious, there are a few narrative sentences |
| 6 points: there is a more complete statement sentence, can appear normal sentence pattern, misspellings still |
| 7 points: smooth, may be endless, on the basis of 6 points can have syntax and rhythm similar to the Chinese phoneme strange language, accompanied by different phoneme misnomer and new word disease |
| 8 points: smooth, sentences are often complete, but can not be related to the subject, there are obvious difficulties in finding words and back-and-forth, there are semantic errors, there can be semantic oddities |
| 9 points: Most of them are complete with the subject of the sentence, occasionally and wrong words, find words some difficult, but some pronunciation errors |
| 10 points: Sentences have normal length and complexity, no definite slowness, hibidness or difficulty in pronunciation, no missteptics |

| **2. Listen to the comprehension check**  **A. Answer yes or no (60 points, 3 points revised)** | | | | |
| --- | --- | --- | --- | --- |
| I. Questions, answers, expressions and ratings |  |  | **3 points** |  |
| issue | The correct answer | Expression | **Score** | Speech characteristics |
|  |  | Speech Hands, eyes closed |  |  |
| (1) Is your name Zhang Minghua? | not |  |  |  |
| (2) Is your name Li Fei? | not |  |  |  |
| (3) Is your name (the patient's real name)? | be |  |  |  |
| (4) Do you live in Urumqi? | not |  |  |  |
| (5) Do you live in (the address where the patient lives)? | be |  |  |  |
| (6) Do you live in Zhengzhou? | not |  |  |  |
| (7) Are you a man (female)? | be |  |  |  |
| Are you a doctor? | not |  |  |  |
| (10) Is there a light in this room? | be |  |  |  |
| (11) Is the door closed? | be |  |  |  |
| (12) Is this a hotel? | not |  |  |  |
| (13) Is this a hospital? | be |  |  |  |
| (14) Are you wearing red pajamas? | not |  |  |  |
| (15) Can paper burn in fire? | be |  |  |  |
| (16) Is March ahead of June? | be |  |  |  |
| (17) Can bananas be eaten without peeling? | not |  |  |  |
| (18) Is it snowing in July? | not |  |  |  |
| (19) Is a horse bigger than a dog? | not |  |  |  |
| (20) Do you cut the grass with an axe? | not |  |  |  |
| Score |  |  |  |  |

**B. Listening to the word recognition (one point per word, a total of 60 points, after correction is still 1 point, refers to 2 things above 0 points)**

| II. Content | | | | |
| --- | --- | --- | --- | --- |
| (1) Physical  cup  match  pencil  Flowers (flowers, plastic flowers, paper flowers)  comb | (2) Drawn objects  match  cup  comb  screwdriver  pencil  flower | (3) Shape  square  triangle  rotundity  arrowhead  cross  cylinder | (4) Pinyin letters  J  F  D  K  M  D | (5) Numbers  5  61  500  1867  32  5000 |
| (6) Color  blue  palm tree  red  green  yellow  black | (7) Furniture  window  chair  desk  table lamp  door  ceiling | (8) Body parts  ear  nose  eye  chest  neck  cheek | (9) Fingers, etc  thumb  ring finger  index finger  little finger  middle finger  Right | (10) The left and right parts of the body  Right shoulder  Left knee  Left ankle  right hand  left elbow  right cheek |
| Score |  | | | |

**C. Successive instructions (out of 80 points) are divided according to the completion steps**

| II Instructions and ratings | Score | Score |
| --- | --- | --- |
| (1) Raise your hand | 2 |  |
| (2) Close your eyes | 2 |  |
| (3) Point to the chair | 2 |  |
| (4) Point first to the window ^(2)^and then to the door ^(2).^ | 4 |  |
| (5) Point to pen ^(2)^ and book ^(2).^ | 4 |  |
| (6) With a pen ^(4)^ fingering ^(4).^ | 8 |  |
| (7) Use a book ^(4)^ to refer to the pen ^(4).^ | 8 |  |
| (8) Use a pen ^(4)^ to comb ^(4).^ | 8 |  |
| (9) Use the book ^(4)^ to comb ^(4).^ | 8 |  |
| (10) Put the pen ^(4)^ on top of the book ^(6)^ and give it to me ^(4).^ | 14 |  |
| (11) Place the comb ^(5)^ on the other side of the pen ^(5)^ and turn the book ^(5)^ over ^(5).^ | 20 |  |

**3. Retelling of the check (up to 100 points, phoneme error or misalignment deduction of 1 point)**

|  | | | | |
| --- | --- | --- | --- | --- |
| The title | issue | Out | Score | Speech characteristics |
| （1） | bed | 2 |  |  |
| （2） | nose | 2 |  |  |
| （3） | pipe | 2 |  |  |
| （4） | window | 2 |  |  |
| （5） | banana | 2 |  |  |
| （6） | snowball | 4 |  |  |
| （7） | forty | 4 |  |  |
| （8） | Percentage | 6 |  |  |
| （9） | Sixty-two-five | 10 |  |  |
| （10） | The bell is ringing | 8 |  |  |
| （11） | He won't come back | 10 |  |  |
| （12） | Master is very happy | 10 |  |  |
| （13） | A wild gun | 8 |  |  |
| （14） | If or however | 10 |  |  |
| （15） | Pack 6 bottles of paint in my box | 20 |  |  |
| Score |  | | | |

**4. Naming check (60 points, 3 points, 2 points for phoneme error, the latter plus 1 point for haptic)**

**A. Name an object**

|  | | | | |
| --- | --- | --- | --- | --- |
| III. Content records | | | | |
| object | react | Tactile tips | Phoneme tips | Score |
| (1) Guns |  |  |  |  |
| (2) Ball |  |  |  |  |
| (3) Knife |  |  |  |  |
| (4) Cup |  |  |  |  |
| (5) Pin |  |  |  |  |
| (6) Hammer |  |  |  |  |
| (7) Toothbrush |  |  |  |  |
| (8) Eraser (for brushing pencil words) |  |  |  |  |
| (9) Padlock |  |  |  |  |
| (10) Pencil |  |  |  |  |
| (11) Screwdriver |  |  |  |  |
| (12) Key |  |  |  |  |
| (13) Paper clips |  |  |  |  |
| (14) Pipe |  |  |  |  |
| (15) Comb |  |  |  |  |
| (16) Rubber band |  |  |  |  |
| (17) Tablespoon |  |  |  |  |
| (18) Transparent film |  |  |  |  |
| (19) Fork |  |  |  |  |
| (20) Matches |  |  |  |  |
| Score |  | | | |

**B. Spontaneous naming (1 minute Animals up to 20 points 1 point for each uttered).**

**C. Complete sentence (10 points, 1 point misnome)**

|  | | | |
| --- | --- | --- | --- |
|  | | | |
| III. Sentences and answers | | | |
| sentence | answer | Rating 2 | Speech characteristics |
| (1) Grass is... target | green |  |  |
| (2) Sugar is... target | Sweet or white |  |  |
| (3) Roses are red, violets are... target | Blue purple |  |  |
| (4) They fight like cats and... The same | dog |  |  |
| (5) Laba is in the lunar calendar ... month | Dec |  |  |
| Score |  | | |

**D. Reaction naming (10 points, 1 point misnome)**

|  | | | |
| --- | --- | --- | --- |
|  | | | |
| QUESTIONS and answers | | | |
| issue | answer | Rating 2 | Speech characteristics |
| 1 What do you write with? | A pen or pencil, brush |  |  |
| (2) What color is the snow? | white |  |  |
| 3 How many days a week? | 7 days |  |  |
| (4) Where does the nurse work? | hospital |  |  |
| (5) Where do you buy stamps? | Post offices, shops |  |  |
| Score |  | | |

**Aphasia Merchant (AQ) calculation method:**

**AQ s (Spontaneous Speech Score and Listening Comprehension Score ÷20 scrime score ÷10 sniter score ÷10) ×2**

WAB rating for major types of aphasia

| project | fluency | understand | retell | christen |
| --- | --- | --- | --- | --- |
| Completeness  motility  Cortical mixing  Cortical sport  Sensory  Percepary sensation  conductibility  Naming | 1. 4 2. 4 3. 4 4. 4   5—10  5—10  5—10  5—10 | 0—3.9  4—10  0—3.9  4—10  0—6.9  0—6.9  7—10  7—10 | 0—4.9  0—4.9  5—10  8—10  0—7.9  8—10  0—6.9  7—10 | 1. 6 2. 8 3. 6 4. 9 5. 9 6. 9 7. 9   0—9 |

Aphasia Merchant: Type of Patient Aphasia:

**No.4 Upper Extremity Fugl-Meyer Assessment**

Name: Gender, age, disease, illness, medical course,

|  | 0 points | 1 point | 2 points | Month Day | Month Day | Month Day |
| --- | --- | --- | --- | --- | --- | --- |
| I. Upper limbs | | | | | | |
| Seat | | | | | | |
| 1 There is no reflective activity | | | |  |  |  |
| (1) Biceps | Does not cause reflection activity |  | Can cause reflection activity |  |  |  |
| (2) Triceps | ditto |  | ditto |  |  |  |
| 2 flexor co-motion | | | | | | |
| (3) Shoulder lift | It can't be done at all | Partially completed | Fully completed without pause |  |  |  |
| (4) Shoulder back retracts | ditto | ditto | ditto |  |  |  |
| (5) Shoulder outreach ≥ 90 degrees | ditto | ditto | ditto |  |  |  |
| (6) Shoulder spin | ditto | ditto | ditto |  |  |  |
| (7) Elbow flexor | ditto | ditto | ditto |  |  |  |
| (8) After the forearm spins | ditto | ditto | ditto |  |  |  |
| 3 Stretch muscle co-motion | | | | | | |
| (9) Shoulder collection, inner rotation | ditto | ditto | ditto |  |  |  |
| (10) Elbow stretch | ditto | ditto | ditto |  |  |  |
| (11) Before the forearm spins | ditto | ditto | ditto |  |  |  |
| 4 Activities accompanied by collaborative movement | | | | | | |
| (12) Tactile lumbar spine | There was no obvious activity | The hand can only cross the front of the armrant backwards | Can go smoothly |  |  |  |
| (13) Shoulder joint flexes 90 degrees, elbow straightening | Start with an immediate outreach of the arm or a flexion of the elbow | Shoulder outreach or elbow flexion as it approaches the specified position | Can be completed smoothly and fully |  |  |  |
| (14) Shoulder 0 degrees, elbow 90 degrees, forearms before and after rotation | Elbows or forearms cannot be twisted | Shoulder, elbow position is correct, basically can spin forward, after | It's done |  |  |  |
| 5 Out of the activities of the concerted movement | | | | | | |
| (15) Shoulder joint outstretched 90 degrees, elbow straight, forearm before rotation | At the beginning the elbow bends and the forearm deviates from the direction and cannot be turned forward | This action can be partially completed or the elbows can be flexed or the forearms cannot be turned during the movement | It's done |  |  |  |
| (16) Shoulder joint front bowed arm over the head, elbow straight, forearm neutral position | Start with elbow flexion or shoulder outreach | Shoulder flexion midway, elbow flexion, shoulder outreach | It's done |  |  |  |
| (17) Shoulder flexion 30 degrees to 90 degrees, elbow straight, forearm spin | The forearm spin is completely unable to perform or the elbow position is incorrect | Shoulder, elbow position is correct, basically can complete the pre-rotation | It's done |  |  |  |
| 6 Reflective radon | | | | | | |
| (18) Check the three reflexes of the biceps, triceps and flexors | At least 2 to 3 reflections are significantly more progressive | One reflection is significantly radoned or at least two reflections are active | Active reflection ≤ 1 and no reflexes |  |  |  |
| 7 wrist stability | | | | | | |
| (19) Shoulder 0 degrees, elbow 90 degrees, wrist back flexor | Cannot back the wrist up to 15 degrees | Can complete the wrist back bend, but can not resist resistance | Applying a slight drag can still keep the back of the wrist flexoring |  |  |  |
| (20) Shoulder 0 degrees, elbow flexion 90 degrees, arm flexion extension | Can not be arbitrarily stretched | The wrist cannot be actively moved within the full joint range | Can be carried out smoothly without stopping |  |  |  |
| 8 elbow straight, shoulder front 30 degrees | | | | | | |
| (21) The back of the wrist is flexed | Cannot back the wrist up to 15 degrees | Can complete the wrist back bend, but can not resist resistance | Applying a slight drag can still keep the back of the wrist flexoring |  |  |  |
| (22) Arm flexion | Can not be arbitrarily stretched | The wrist cannot be actively moved within the full joint range | Can be carried out smoothly without stopping |  |  |  |
| (23) Wrist ring movement | Cannot proceed | Activities are laborious or incomplete | Complete normally |  |  |  |
| 9 fingers | | | | | | |
| (24) Group buckling | Cannot bend | Can bend but not enough | Can be completely active buckling |  |  |  |
| (25) Group stretch | Cannot stretch | Can relax the active flexor of the finger | Fully active stretching |  |  |  |
| (26) Hook grip | The required position cannot be maintained | The grip is weak | Be able to resist considerable resistance |  |  |  |
| (27) Side pinch | Cannot proceed | You can hold a piece of paper with your thumb, but you can't resist pull | Hold the paper firmly |  |  |  |
| (28) Pinch (thumb index finger can hold a pencil) | Not at all | The pinch is weak | Can resist considerable resistance |  |  |  |
| (29) Cylindrical grip | Same (26) | Same (26) | Same (26) |  |  |  |
| (30) Spherical grip | ditto | ditto | ditto |  |  |  |
| 10 Coordination ability and speed (finger finger nose test 5 times in a row) | | | | | | |
| (31) Tremors | visible tremors | Mild tremors | No tremors |  |  |  |
| (32) Distance barrier | Obvious or irregular barriers to discernment | Mild or regular barrier to distance | No discernment barrier |  |  |  |
| (33) Speed | 6 seconds longer than the healthy side | The healthier side is longer for 2 to 5 seconds | The difference between the two sides < 2 seconds |  |  |  |

**No.5 Action Research Arm Test**

0 points: No action can be taken

1 point: Can be partially completed

2 points: can be completed, but the movement is not smooth or there is a time delay

3 points: Can be completed normally

| **Evaluate the content** | | | Limited time (seconds) | Score |
| --- | --- | --- | --- | --- |
| **scratch** | 1 | Grab a 2.5cm^3^ piece of wood | 3.6 |  |
|  | 2 | Grab a^5cm 3^ piece of wood | 3.5 |  |
|  | 3 | Grab a 7.5cm^3^ block of wood | 3.9 |  |
|  | 4 | Grab a ball with a diameter of 7.5cm | 3.8 |  |
|  | 5 | Grab a 10×25×1cm stone | 3.6 |  |
|  | 6 | Grab a 10cm^3^ piece of wood | 4.2 |  |
| **grip** | 1 | Hold a tube with a diameter of 2.25cm | 4.2 |  |
|  | 2 | Hold a tube 1cm in diameter | 4.3 |  |
|  | 3 | Connect the 3.5cm diameter washer to the bolts on the table | 4 |  |
|  | 4 | Pour water from one glass into another | 7.9 |  |
| **pinch** | 1 | Pinch the 1.5cm diameter ball with your index finger and thumb | 3.8 |  |
|  | 2 | Pinch the 1.5cm diameter ball with your middle finger and thumb | 3.8 |  |
|  | 3 | Pinch the 1.5cm diameter ball with your ring finger and thumb | 4.1 |  |
|  | 4 | Squeeze the 0.6cm diameter ball with your index finger and thumb | 4 |  |
|  | 5 | Pinch the 0.6cm diameter ball with your middle finger and thumb | 4.1 |  |
|  | 6 | Pinch a ball with a .6cm diameter with your ring finger and thumb | 4.4 |  |
| **Big movement** | 1 | Touch your mouth with your hands | 2.4 |  |
|  | 2 | Put your hands on your head | 2.7 |  |
|  | 3 | Put your hands behind your head | 2.7 |  |
| **Score** | | | |  |

**No.6 Activities of Daily Living**

Corbe: Bed Number: Hospital Number:

| Name: Gender: Age: (year/month) Home address/phone: | | | | | | | |
| --- | --- | --- | --- | --- | --- | --- | --- |
| Clinical diagnosis: | | | | | | | |
| Item | Ratings are accurate | score  date | | score  date | | score  date | |
| Stool | 0 - Incontinence or coma; 5 - Occasional incontinence (once a week); 10 - Controlled< |  |  |  |  |  |  |
| Urinating | 0 - Incontinence, coma or need to conduct urine; 5 - Occasional incontinence (once a week every 24h1);<>  10 - Can control |  |  |  |  |  |  |
| Retouching | 0 - Need help; 5 - Wash face, comb, brush your teeth, shave your beard independently |  |  |  |  |  |  |
| Such as toilets | 0 - Dependency; 5 - Need partial help; 10 - Self-care |  |  |  |  |  |  |
| Eat | 0 - dependence; 5 - some help (e.g. cutting bread, buttering, sandwiching, serving, etc.);  10 - All self-care |  |  |  |  |  |  |
| Transfer | 0 - Total dependence (needs more than 2 people for help); 5 - requires 2 people or 1 strong, skilled person to help or guide;  10 - Requires a little help; |  |  |  |  |  |  |
| Live  (Walking) | 0 - Unable to move; 5 - Independent in a wheelchair; 10 - 1 person help required (physical or verbal guidance); 15 - Independent walking (assistive devices available) |  |  |  |  |  |  |
| Dressing | 0 - Dependency; 5 - Half help required; 10 - Self-care (buttons, switch zippers, shoes and bras) |  |  |  |  |  |  |
| Up and down  Staircase | 0 - No; 5 - Need help (physical or verbal guidance); 10 - Self-care |  |  |  |  |  |  |
| Take a bath | 0 - Dependency; |  |  |  |  |  |  |
| Score: | |  |  |  |  |  |  |
| Degree of competency deficiency: | |  |  |  |  |  |  |
| Raters: | |  |  |  |  |  |  |

ADL capacity defects: 0 to 20 - very serious functional defects; 25 to 45 - severe functional defects;

50 to 70 - moderate functional defects; 75 to 95 - mild functional defects;

100 - ADL self-care

**No.7 Consolidate Summary Tables**

| project | score | The evaluation date |
| --- | --- | --- |
| BDAE |  |  |
| WAB - Spontaneous Language feature |  |  |
| WAB-Understanding |  |  |
| WAB-Retelling feature |  |  |
| WAB-Naming feature |  |  |
| WAB-AQ |  |  |
| Fugl-meyer (upper limb) scores |  |  |
| ARAT score |  |  |
| ADL score |  |  |

Assessor's Signature: ________

Patient's Signature: ________

Family member's Signature: _______
